# What Is the Importance of Electrocardiography in the Routine Screening of Patients with Repaired Tetralogy of Fallot?

**DOI:** 10.3390/jcm10194298

**Published:** 2021-09-22

**Authors:** Paulina Lubocka, Robert Sabiniewicz

**Affiliations:** Department of Paediatric Cardiology and Congenital Heart Disease, Medical University of Gdańsk, 80-952 Gdańsk, Poland; info@gumed.edu.pl

**Keywords:** tetralogy of Fallot, electrocardiography, multiparametric magnetic resonance imaging, pulmonary valve insufficiency, right bundle branch block, heart rate variability

## Abstract

Background: In patients following complete repair of the tetralogy of Fallot, the duration of the QRS complex is associated with the size and mechanical function of the right ventricle, which are contemporarily assessed by cardiac magnetic resonance (CMR). Methods: 38 patients aged 18.0–54.9 years (median age 24.9 years) who had undergone complete repair of the tetralogy of Fallot were examined using CMR and concomitant 24 h ambulatory electrocardiography monitoring. We used statistical analysis to investigate the correlations between electrocardiographic parameters (heart rate, HR; PQ interval, PQ; QRS duration, QRS; and corrected QT interval, QTc) and CMR results (right ventricular ejection fraction, RVEF; right ventricular end-diastolic volume index, RVEDVI; and right ventricular end-systolic volume index, RVESVI) for patients after early and late repair. Results: The ECG-based parameters were not correlated with time since repair. There were significant correlations between QRS duration and RVEF (r = −0.61), RVEDVI (r = 0.56), and RVESVI (r = 0.54) for early operated patients but not for late-operated patients. No other substantial correlations were reported. Conclusion: Despite its role in screening for arrhythmias, electrocardiography has a limited role as a predictor of morphology and function of the right ventricle in patients after repair of the tetralogy of Fallot.

## 1. Introduction

The pathophysiology of a repaired tetralogy of Fallot (rTOF) is an interplay between the anatomy of the heart and its electrical and mechanical function. Persistent pulmonary regurgitation results in dilatation of the right ventricle (RV) and fibrosis. Consequently, the diastolic compliance of RV decreases, leading to the elevation of end-diastolic pressure in RV and a further increase in the pulmonary regurgitation fraction. On the other hand, the scar surrounding the RV outflow tract (RVOT) patch, combined with impaired histological structure of the RV wall due to volume and pressure overload, are a substrate for conduction delay and ventricular arrhythmias. As a result, the typical electrocardiogram (ECG) features in rTOF are wide QRS complexes with a right bundle branch block (RBBB) pattern, and arrhythmias are among the most frequent causes of death in patients who survive rTOF, together with heart failure and myocardial infarction [1,2].

Routine screening of these patients therefore includes a standard ECG and echocardiography. Additionally, a 24 h Holter ECG recording is performed periodically to screen for asymptomatic arrhythmias. Cardiac magnetic resonance (CMR) is also performed, which is currently the gold standard for volumetric and functional assessment of the RV. It is recommended that CMR should be performed before therapeutic interventions such as valve replacement or balloon valvuloplasty, and routinely every 2–3 years from adolescence in rTOF patients [3,4,5].

Given that the electrical anomalies are partly explained by RV enlargement, in the present study, we aimed to investigate the utility of ECG in evaluating RV morphology and function in rTOF patients by investigating the correlation between results of concomitant ECG diagnostics with Holter monitor and CMR.

## 2. Materials and Methods

We performed a retrospective study on patients who were followed up in our unit following complete TOF repair. The inclusion criteria were primary diagnosis of tetralogy of Fallot, regular screening visits in our unit, at least one CMR performed between February 2013 and July 2021, age ≥ 18 years at time of CMR, and sinus rhythm on ECG. Where a patient had more than one CMR examination during the timeframe, only the most recent examination was taken into consideration.

CMR was performed on a 1.5 T scanner (MAGNETOM Aera, Siemens Healthcare, Erlangen, Germany). All subjects underwent routine cine imaging for functional assessment of the left ventricle (LV and RV using a balanced steady-state-free-precession sequence (bSSFP). Three standard long axis cines as well as short axis and transverse cine stacks were obtained every 10 mm (slice thickness 7 mm) from the base to apex and from the level of the diaphragm up to the level of the major arteries. The in-plane resolution was typically 1.4 × 1.4 mm. Next, 2D phase-contrast gradient echo (GRE) sequences were used to measure flow in the main, right, and left pulmonary arteries and in the ascending aorta, carefully checking for aliasing. Then, late gadolinnium enhancement (LGE) images were acquired 8–15 min after contrast administration (gadobutrol 0.1 mmol/kg) using both segmented and single-shot IR-GRE sequence with inversion time adjusted as needed. A simplified scoring system was applied to assess LGE. Considering that the foci of LGE in rTOF patients can be found in four main sites (RVOT, interventricular septum—around the ventricular septal defect (VSD) patch, at the insertion points of the RV on to the LV, and in the LV free wall), the patients in whom LGE was limited to VSD and RVOT were assigned LGE value of 1, 2 points if a focus was found at the insertion points, and 3 points for LGE outside the insertion points.

Additionally, data from 24 h Holter ECG recordings obtained as a part of concurrent ambulatory visits were collected. The time interval from Holter ECG to CMR was less than 12 months. After general analysis and manual correction of the recordings, automatic measurements were collected including average heart rate (HR) and heart rate variability (HRV) as well as the percentages of supraventricular and ventricular arrhythmias in the recording. The following indices of HRV were calculated: the average of all five-minute standard deviations of NN intervals (ASDNN), the standard deviation of all five-minute averages (SDANN), the standard deviation of all NN intervals (SDNN), and the square root of the mean squared differences of successive NN intervals (RMSSD). Interval measurements (PQ and QRS) were obtained from a fragment of ECG, in which the HR was rhythmic and equal with the average HR from the 24 h recording. After the formation of a five-electrode 12-lead vector-based ECG reconstruction (EASI), the PQ interval was measured semi-automatically in lead II and the QRS was measured in a lead where its length from the onset to the crossing with the isoelectric line was maximal. All of the interval measurements were obtained by a single investigator, blinded to the results of CMR. The average of two independent measurements was taken for statistical analysis. Considering the importance of QRS duration in risk stratification and its potential dependence on momentary HR, we decided to correct the QRS for HR according to a formula created by previous authors [6]:

QRSc = QRS + 0.0125 × (1000 − RR),

where QRSc—corrected QRS duration; QRS—measured QRS duration; and RR—mean RR interval.

Ventricular and supraventricular arrhythmias during the recording were recognized where the number of inappropriate beats exceeded 0.1% of the 24 h recording. Non-sustained ventricular tachycardia (nsVT) was diagnosed as an episode of three or more consecutive ventricular beats at a rate of greater than 100 beats per minute with a duration of less than 30 s.

The data were expressed as mean +/− SD. Comparisons between independent groups were made with Student’s *t*-test after testing for normality using the Shapiro–Wilk method. Where the distribution of a variable differed from normal, the Mann–Whitney U test was used. Relationships between continuous variables were analyzed using Spearman’s correlation coefficient, and Pearson’s chi-squared test was used for testing relationships between categorical variables. For all analyses, a value of *p* < 0.05 was considered statistically significant. Statistical analysis was performed using Statistica software, version 13 (TIBCO Software Inc., 2017, Palo Alto, CA, USA).

## 3. Results

**Figure 1:**
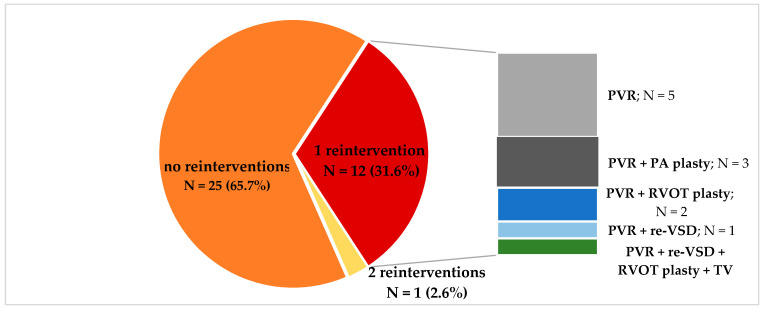
Surgical reinterventions after TOF repair; PVR = pulmonary valve replacement, PA = pulmonary artery; RVOT = right ventricular outflow tract; re-VSD = residual postsurgery ventricular septal defect.

A total of 39 patients met the inclusion criteria, out of whom one was excluded due to insufficient quality of the CMR results. The final group therefore consisted of 38 subjects (11 male) aged 18.0–54.9 years (median age 24.9 years) who had undergone a complete TOF repair at the age of 16 days to 15.1 years (median age 2.4 years). A palliative procedure had been performed in 10 of them (26.3%) prior to complete correction. Fifteen of the patients (39.5%) had their repair within the first 12 months of life (early repair), and the remaining 23 patients (60.5%) had the procedure later in life (late repair). RVOT was reconstructed in 16 subjects (42.1%) with a transannular patch (TAP) and in 6 of them (26.1%), RVOT was reconstructed with a homograft. In the remaining 16 patients (42.1%), pulmonary comisurotomy was performed along with infundibulectomy and non-transannular patch insertion (Table 1). The time from surgery to CMR was 15.1–42.3 years (median time 22.7 years). Within that time, 17 patients (32.7%) had at least one surgical reintervention, primarily comprising pulmonary valve replacement. Figure 1 presents the details regarding the surgical history of the patients.

In terms of ECG results, 28 patients (80.0%) had RBBB and 7 (20.0%) had incomplete RBBB (IRBBB) features; 12 patients had a single ventricular arrhythmia on their 24 h recording, 21 had a benign supraventricular arrhythmia, and four had at least one episode of nsVT. All patients who developed nsVT had undergone late repair: a 45-year-old male with TAP at the age of 13.1 years, a 40-year-old patient with nontransannular patch through ventriculotomy at the age of 3.7 years, a 36-year-old operated with the same method at the age of 8.5, and a 35-year-old with pulmonary homograft implantation at 8.2 years. QRS prolongation was observed in three of them, whereas the remaining one had normal QRS duration. The relative intraobserver variability of the two QRS measurements was 2.0% (6.1 ms) on average. Table 2 summarizes the main characteristics of the study population.

**Figure 2:**
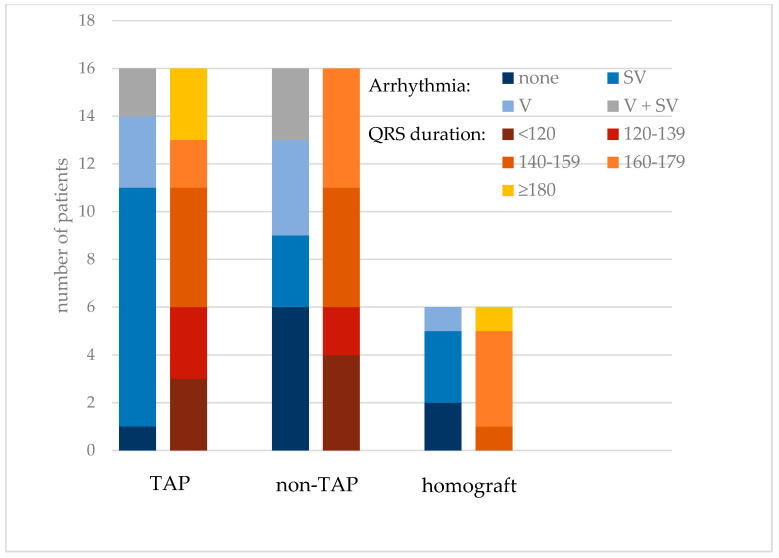
The presence of ventricular and supraventricular arrhythmia along with QRS duration in rTOF patients according to the type of RVOT reconstruction; non-TAP = reconstruction of the right ventricular outflow tract without transannular patch; TAP = transannular patch insertion; SV = supraventricular arrhythmia >0.1% of the recording; V = ventricular arrhythmia >0.1% of the recording; V + SV = both ventricular and supraventricular arrhythmia registered.

Considering the possible discrepancy between patients after early and late repair, we performed a comparative analysis of these two groups regarding all of the analyzed variables. The patients after late correction were older at the screening visit (*p* < 0.001); however, there were no substantial differences in terms of indexed RV measurements or the ECG parameters. The type of surgical repair had no substantial influence on the presence of ventricular arrhythmia (*p* = 0.239), on the percentage of ventricular beats in the Holter recording (*p* = 0.553), or on the QRS duration (*p* = 0.093) (Figure 2).

**Figure 3:**
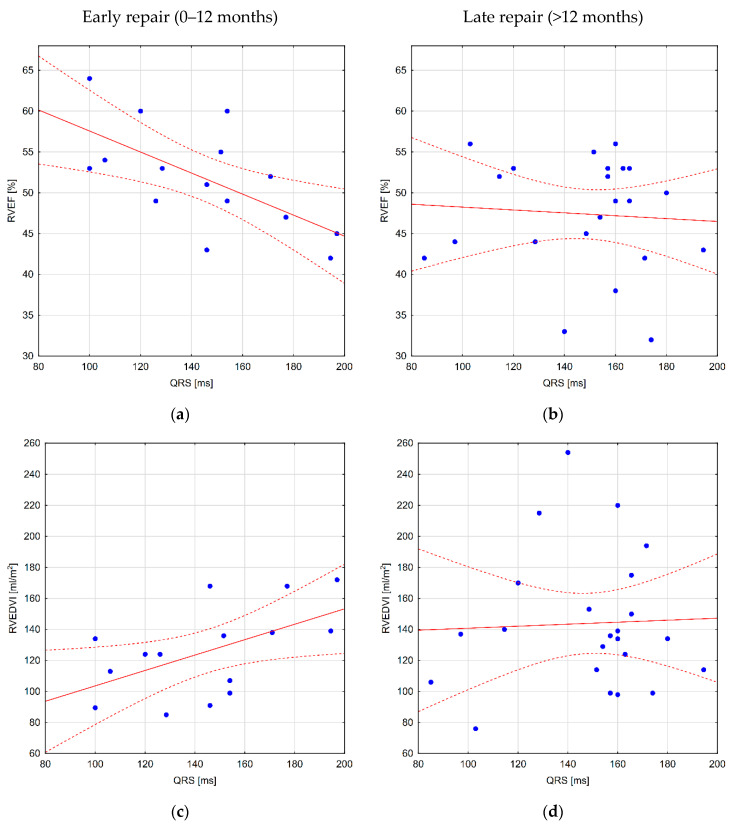

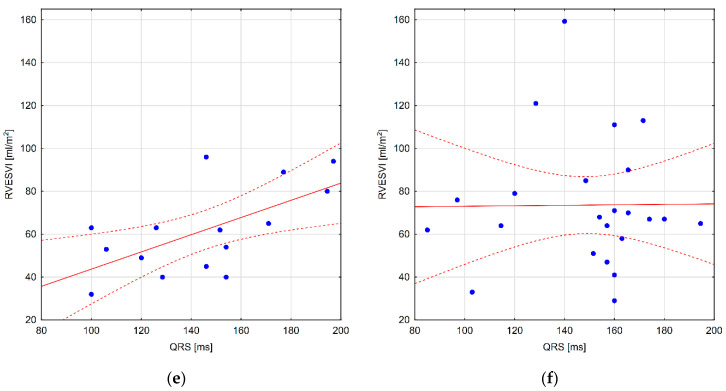
Scatter charts showing the relationship between QRS duration and the CMR parameters: (**a**) right ventricular ejection fraction (RVEF) in patients after early repair; the Spearman correlation coefficient is significant (r = −0.61); (**b**) RVEF in patients after late repair; no substantial correlation was observed (*p* > 0.05); (**c**) right venricular end-diastolic volume index (RVEDVI) in patients after early repair; the Spearman correlation coefficient is significant (r = 0.56); (**d**) RVEDVI in patients after late repair; no substantial correlation was observed (*p* > 0.05) (**e**) right venricular end-systolic volume index (RVESVI) in patients after early repair; the Spearman correlation coefficient is significant (r = 0.54); (**f**) RVESVI in patients after late repair; no substantial correlation was observed (*p* > 0.05). Solid line represents linear regression and the dotted lines stand for regression tolerances.

In the analysis of correlations between the results of 24 h Holter ECG and CMR for the whole group, PQ duration corresponded with a right ventricular ejection fraction (RVEF) (r = −0.34), and longer QRS duration was associated with a higher right ventricular end-systolic volume index (RVESVI) (r = 0.32). The measurements of HR variability were not associated with RV size; however, substantial negative correlations were found between two of the HRV indices and pulmonary ejection fraction. Furthermore, there was a substantial correlation of pulmonary regurgitation fraction (PRF), with RVEDVI and RVESVI but not with the ECG parameters. Similarly, older age at CMR was associated with greater RVEDVI and RVESVI but not with greater QRS duration (Table S1). The analysis for distinct subgroups (early versus late correction) revealed significant correlations between QRS duration and RV size in patients after early repair (Figure 3). No additional correlations were found (Tables S2–S4).

There were no substantial differences in QRS duration (*p* = 0.448) according to the assigned LGE score. The data regarding LGE analysis can be found in the Supplementary Materials Figures S1 and S2.

## 4. Discussion

The results suggest that hemodynamic and electrical functions of the RV do not correlate precisely with each other in all rTOF patients. Among the ECG parameters considered in this study, solely the QRS duration in early operated patients was affected by RV size, as estimated by CMR. This finding implies that QRS prolongation in TOF patients results from a combination of factors of varying impact, depending on the time since repair. Noticeably, PRF, which is known to be a major determinant of RV size in patients with corrected TOF, was not correlated with QRS duration in any of the groups.

Widening of the QRS complex with a RBBB pattern on the ECG, is a feature observed in the vast majority of patients with rTOF [7,8]. Typically, the conduction delay first occurs immediately after surgery as a result of damage to the right bundle branch (RBB) at various levels [9]. Following surgery, the delay gradually increases with ongoing RV dilatation and remodeling; thus, further QRS prolongation is observed [10]. In multiple prospective and retrospective studies, patients with longer QRS complexes and greater rate of elongation have been shown to be at higher risk of ventricular arrhythmias and sudden cardiac death [11,12,13].

Similarly, data from CMR deliver important information in terms of prognosis and risk stratification. According to current guidelines, severely impaired RV and LV function is associated with life-threatening ventricular arrythmias, such as polymorphic ventricular tachycardia and ventricular fibrillation [5]. In a cohort of 100 patients examined with CMR at least 10 years after TOF repair, reduced LVEF and RVEF were associated with impaired clinical status [14] and adverse outcomes including death and nsVT [15]. Additionally, Beurskens recently reported that higher RVEDV and RVESV, together with lower RVEF, were found to be independent predictors of ventricular arrhythmia in a group of 423 rTOF patients [16].

The relation between RV morphology and QRS duration was also investigated previously. Abd El Rahman et al. showed a positive correlation between RVEDV and RVESV absolute values as estimated by 3D echocardiography with QRS duration [17] on an age-diverse group of patients. Uebing et al. [18] attributed the QRS widening to RVOT disease and showed that QRS duration corresponded with contraction delay of this area as measured by echocardiography.

Furthermore, pulmonary valve replacement has been found not only to limit further elongation of QRS [19] but also to trigger QRS shortening via a reduction in RV volume [20]. On the contrary, a recent analysis of QRS duration in rTOF patients after pulmonary valve replacement has shown that the process of QRS prolongation proceeds despite intervention but appears to be more steady in subjects with a smaller RV size at the time of operation [21]. To our knowledge, the differences between the impact of RV volume on QRS duration have not been investigated for early and late repaired TOF patients separately.

Although RV dilatation is the most remarkable sign of volumetric overload and the major determinant of QRS prolongation in rTOF subjects, other concomitant factors that contribute to the impairment of RV mechanics have been identified. Among them, RV fibrosis, ischemia, formation of aneurysms, and RV wall stress are the most important [3]. Notably, time of repair affects the histological structure of RV wall in TOF patients, and it has been reported that patients who undergo the surgery at the age of 4 years or more were more likely to develop cellular hypertrophy, interstitial fibrosis, and endocardial thickening in the RVOT tissue resected during the procedure [22]. Considering the difference in baseline structure of RV wall in early and late repaired TOF as well as the irreversibility of myocardial fibrosis [23], we can expect differences in RV mechanics in the two groups and more pronounced intraventricular conduction delay in late repaired patients. The absence of a correlation between QRS duration and RV size additionally supports this theory as well as the higher prevalence of LGE sites among late repaired subjects (Figure S2).

rTOF patients were found to have lower HRV parameters obtained from 24 h Holter recording in comparison with healthy controls [24]. In our study, we did not observe any clear relationship between results of CMR and HRV. There were several low-grade correlations between HRV indices and PRF, which require verification with a bigger sample number.

Some authors claim that the morphology of the QRS complex has a superior role over its duration in risk stratification in patients with repaired TOF [7,9,25]. Apart from simple RBBB pattern, QRS fragmentation (fQRS) has been described, which is defined as the presence of additional R waves in two of more contiguous leads. fQRS was found to correlate with RV size assessed by CMR [23] and was found to be an independent predictor of ventricular arrhythmia [8] in TOF patients. In our study, the fQRS was not analyzed using the 24 h ECG recordings. Despite the EASI reconstruction being shown to have an excellent agreement with 12-lead ECG in interval measurements [26], it has not been validated for evaluation of discrete anomalies in QRS morphology.

Considering that the rapid advancement in cardiac surgery over the past decade has enabled TOF repair in younger children, patients with early and late TOF repairs differ by age. We cannot exclude that the discrepancy between them in terms of ECG–CMR correlation results from age difference at the time of screening rather than from the time of repair. Since our oldest rTOF patients operated on in infancy are currently young adults, a comparison of age-matched groups is, to date, infeasible. Our study demonstrated no significant differences in QRS duration and presence of ventricular arrhythmia in regard to the surgical technique. Considering that the sample number was relatively small for this analysis, we leave this issue for further investigation in future studies.

## 5. Conclusions

In conclusion, the ECG parameters have a poor predictive value for assessing the RV morphology and function in rTOF patients. In the era of advanced image diagnostics, QRS prolongation has limited sensitivity as a marker of RV enlargement and cannot be adequately used in clinical practice as a single parameter. HRV analysis from a 24 h ECG recording provides no conclusive data on the hemodynamic status of rTOF patients either. Considering the complexity of mechanoelectrical interactions after TOF repair, a regular multimodal assessment of these patients is necessary for adequate risk stratification. The prognostic factors encompass a patient’s surgical history, and ventricular dimensions and function assessed by CMR, together with the ECG parameters. Importantly, Holter ECG monitoring is a valuable tool in screening for heart rhythm disturbances, which are responsible for a substantial proportion of deaths in this population.

## Figures and Tables

**Table 1:** Clinical data regarding patients after early and late repair; B-T—Blalock-Taussig shunt; CMR—cardiac magnetic resonance, M—median; RVOT—right ventricular outflow tract; TAP—transannular patch; and nonTAP—nontransannular RVOT patch.

	Early Repair (N = 15)	Late Repair (N = 23)
Palliative procedure prior to complete repair	1 patient (6.7%):	9 patients (34.8%):
	B-T at 3 months of age followed by complete repair at 8 months	B-T; N = 6
		Waterson shunt followed by B-T; N = 1
		B-T followed by modified B-T; N = 1
		Age at palliation: 0.4 to 6 years (M = 1.7)
		Age at repair: 2.4 to 15.1 years (M = 8.2)
Type of RVOT reconstruction	TAP; N = 5 (33.3%)	TAP; N = 11 (47.8%)
	nonTAP; N = 7 (46.6%)	nonTAP; N = 9 (39.1%)
	homograft; N = 3 (20.0%)	homograft; N = 3 (13.0%)
Age at repair without paliation (years)	0.04–1.0 (M = 0.77)	1.4–14.9 (M = 3.3)
Type of RVOT at CMR	TAP; N = 3 (20.0%)	TAP; N = 8 (34.8%)
	nonTAP; N = 4 (26.7%)	nonTAP; N = 8 (34.8%)
	homograft; N = 8 (53.3%)	homograft; N = 7 (30.4%)
	Early repair (N = 15)	Late repair(N = 23)
Palliative procedure prior to complete repair	1 patient (6.7%):	9 patients (34.8%):
	B-T at 3 months of age followed by complete repair at 8 months	B-T; N = 6
		Waterson shunt followed by B-T; N = 1
		B-T followed by modified B-T; N = 1
		Age at palliation: 0.4 to 6 years (M = 1.7)
		Age at repair: 2.4 to 15.1 years (M = 8.2)
Type of RVOT reconstruction	TAP; N = 5 (33.3%)	TAP; N = 11 (47.8%)
	nonTAP; N = 7 (46.6%)	nonTAP; N = 9 (39.1%)
	homograft; N = 3 (20.0%)	homograft; N = 3 (13.0%)
Age at repair without paliation (years)	0.04–1.0 (M = 0.77)	1.4–14.9 (M = 3.3)
Type of RVOT at CMR	TAP; N = 3 (20.0%)	TAP; N = 8 (34.8%)
	nonTAP; N = 4 (26.7%)	nonTAP; N = 8 (34.8%)
	homograft; N = 8 (53.3%)	homograft; N = 7 (30.4%)

**Table 2:** Descriptive characteristics of the population. For variables which were not normally distributed (‡), the median was given instead of mean and standard deviation; * = significant difference between patients after early and late repair. CMR—cardiac magnetic resonance; LVEF—left ventricular ejection fraction; RVEF—right ventricular ejection fraction; RVEDVI—right ventricular end-diastolic volume index; RVESVI—right ventricular end-systolic volume index; RVSVI—right ventricular stroke volume index; PRF—pulmonary regurgitation fraction, ECG—electrocardiography; HR—heart rate; ASDNN—average of all 5 min standard deviations of NN intervals; SDANN—standard deviation of the all five-minute averages; SDNN—standard deviation of all NN intervals; RMSSD—square root of the mean squared differences of successive NN intervals; V_ar—ventricular arrhythmia; SV_ar—supraventricular arrhythmia; nsVT—non-sustained ventricular tachycardia.

	All (N = 38)				Early Repair (N = 15)			Late Repair (N = 23)		
	M ± SD		(min–max)		M ± SD		(min–max)	M ± SD		(min–max)
**Age at CMR (years)** * (*p* < 0.001)	24.9 ‡		(18.0–54.9)		19.7 ± 2.0		(18.0–25.1)	32.5 ‡		(18.3–54.9)
Age at repair (years) * (*p* < 0.001)	2.4 ‡		(0.04–15.1)		0.7 ‡		(0.04–1.0)	5.8 ± 4.3		(1.4–15.1)
**CMRI**										
**LVEF (%)**	61.0 ‡		(37.0–72.0)		61.9 ± 4.4		(50.0–69.0)	59.2 ± 8.4		(37.0–72.0)
**RVEF (%)**	49.1 ± 6.9		(32.0–64.0)		51.8 ± 6.3		(42.0–64.0)	47.4 ± 6.8		(32.0–56.0)
**RVEDVI (mL/m^2^)**	134.0 ‡		(76.0–275.0)		124.0 ‡		(85.0–172.0)	144.8 ± 46.3		(76.0–275.0)
**RVESVI (mL/m^2^)**	64.5 ‡		(29.0–190.0)		62.0 ‡		(32.0–96.0)	74.9 ± 34.3		(29.0–190.0)
**RVSVI (mL/m^2^)**	65.6 ± 16.7		(32.0–108.0)		64.0 ± 11.1		(45.0–80.0)	66.6 ± 19.7		(31.6–108.0)
**RF (%)**	30.9 ± 17.2		(1.0–59.0)		31.9 ± 18.0		(1.0–56.0)	30.2 ± 17.0		(3.0–59.0)
**ECG**										
**HR (bpm)**	77.1 ± 7.3		(62.0–94.0)		76.4 ± 6.6		(65.0–86.0)	77.5 ± 7.9		(62.0–94.0)
**PQ (ms)**	143.0 ‡		(97.0–257.0)		143.0 ‡		(109.0–177.0)	167.3 ± 46.3		(97.0–257.0)
**QRS (ms)**	146.9 ± 28.9		(85.0–197.0)		146.0 ‡		(100.0–197.0)	148.3 ± 27.8		(85.0–194.5)
**>140 (N, %)**	25, 65.8%				9, 60.0%			16, 69.6%		
**>180 (N, %)**	4, 10.5%				2, 13.3%			2, 8.7%		
**QTc (ms)**	466.8 ± 25.1		(420.0–538.0)		464.5 ± 22.9		(420.0–506.0)	468.2 ± 26.9		(430.0–538.0)
**ASDNN**	63.0 ± 17.2		(19.2–105.8)		68.9 ± 13.1		(39.4–122.5)	60.8 ‡		(19.2–89.6)
**SDANN**	119.5 ± 2.3		(56.0–182.3		131.3 ± 27.9		(79.4–182.3)	111.8 ± 33.1		(56.0–173.8)
**SDNN**	141.7 ± 32.5		(68.1–210.8)		153.9 ± 27.2		(94.3–220.0)	133.8 ± 33.7		(68.1–186.8)
**RMSSD**	60.7 ‡		(16.31–75.5)		66.8 ‡		(25.8–169.2)	66.8 ± 44.3		(16.3–175.5)
**V_ar (N, %)**	12	31.6%			4	26.7%		8	34.8%	
**SV_ar (N, %)**	21	55.3%			9	60.0%		12	52.2%	
**nsVT (N, %)**	4	10.5%			0	0.0%		4	17.4%	

## Supplementary Materials

The following are available online at https://www.mdpi.com/article/10.3390/jcm10194298/s1, Figure S1: Prevalence of late gadolinium enhancement (LGE) sites among early and late repaired TOF patients, Figure S2: QRS duration according to the number of detected late gadolinium enhancement (LGE) sites, Table S1: Spearman correlation coefficients of analyzed variables and time from TOF repair to screening visit, Table S2: Spearman correlation coefficients between ECG and CMR results for all patients, Table S3: Spearman correlation coefficients between ECG and CMR results for patients after early TOF repair, Table S4: Spearman correlation coefficients between ECG and CMR results for patients after late TOF repair.
