# Supplementary material for: What Is the Importance of Electrocardiography in the Routine Screening of Patients with Repaired Tetralogy of Fallot?
Source: J Clin Med. 2021 Sep 22;10(19):4298. doi: 10.3390/jcm10194298 (PMC8509678; doi:10.3390/jcm10194298)
Supplement: Supplementary file 1 [file jcm-10-04298-s001.zip › jcm-1374481-supplementary.pdf]

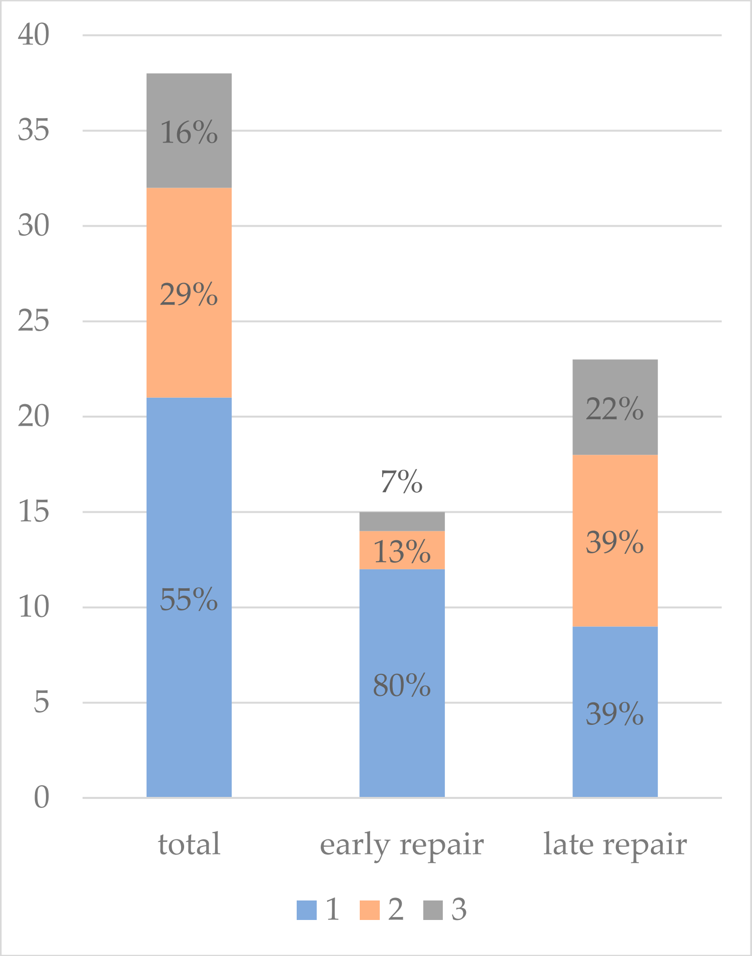


**Figure S1.** Prevalence of late gadolinium enhancement (LGE) sites among early and late repaired ToF patients. Simplified LGE score was statistically higher in patients after late repair (p=0.046); 1 – LGE limited to the sites of VSD and RVOT patches.; 2 – LGE found in the RV insertion points; 2 – LGE outside the RV insertion points.

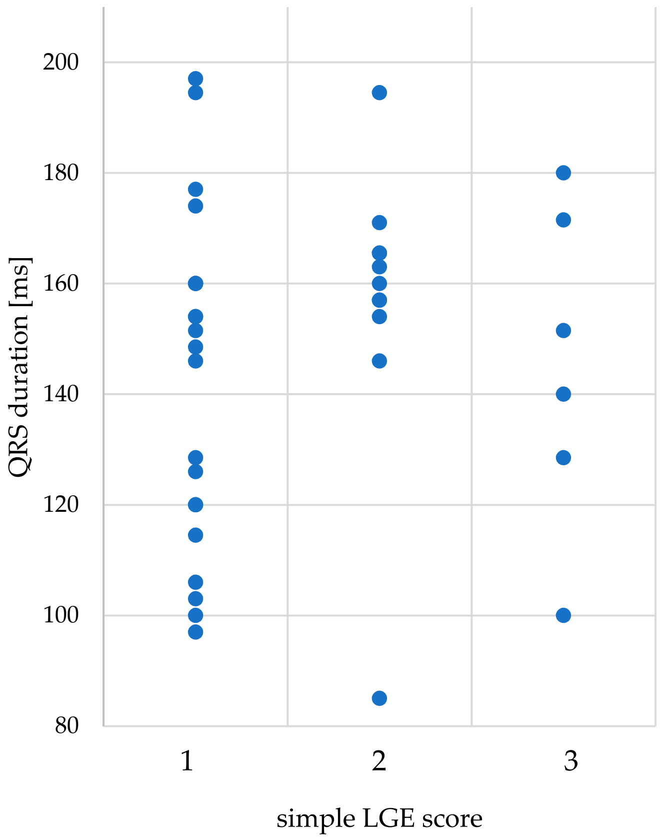


**Figure S2.** QRS duration according to the type of detected late gadolinium enhancement (LGE) sites. Each dot represents one patient; 1 – LGE limited to the sites of VSD and RVOT patches.; 2 – LGE found in the RV insertion points; 2 – LGE outside the RV insertion points.

**Table S1.** Spearman correlation coefficients of analyzed variables and time from ToF repair to screening visit; * = statistically significant results; ECG – electrocardiography; HR – heart rate; ASDNN - average of all 5-minute standard deviations of NN intervals; SDANN - standard deviation of the all five-minute averages; SDNN - standard deviation of all NN intervals ; RMSSD - square root of the mean squared differences of successive NN intervals; CMR – cardiac magnetic resonance; LVEF – left ventricular ejection fraction; RVEF – right ventricular ejection fraction; RVEDVI – right ventricular end-diastolic volume index; RVESVI - right ventricular end-systolic volume index; RVSVI - right ventricular stroke volume index; PRF – pulmonary regurgitation fraction.

| ECG | r | CMR | r |
|---|---|---|---|
| PQ | 0,23 | LVEF | -0,27 |
| QRS | 0,16 | RVEF | -0,24 |
| QTc | 0,15 | RVEDVI | 0,37* |
| HR | 0,14 | RVESVI | 0,40* |
| ASDNN | -0,23 | RVSVI | 0,30 |
| SDANN | -0,14 | PRF | 0,08 |
| SDNN | -0,29 | | |
| RMSSD | -0,31 | | |

**Table S2.** Spearman correlation coefficients between ECG and CMR results for all patients; * = statistically significant results; QRSc - QRS duration corrected for heart rate; HR – heart rate; ASDNN - average of all 5-minute standard deviations of NN intervals; SDANN - standard deviation of the all five-minute averages; SDNN - standard deviation of all NN intervals ; RMSSD - square root of the

mean squared differences of successive NN intervals; LVEF – left ventricular ejection fraction; RVEF – right ventricular ejection fraction; RVEDVI – right ventricular end-diastolic volume index; RVESVI - right ventricular end-systolic volume index; RVSVI - right ventricular stroke volume index; PRF – pulmonary regurgitation fraction.

| | LVEF | RVEF | RVEDVI | RVESVI | RVSVI | PRF |
|---|---|---|---|---|---|---|
| PQ | -0.24 | -0.34* | 0.10 | 0.17 | -0.13 | -0.25 |
| QRS | -0.14 | -0.32* | 0.26 | 0.32* | 0.10 | -0.15 |
| QRSc | -0.13 | -0.31 | 0.26 | 0.32 | 0.10 | -0.15 |
| QTc | -0.12 | -0.06 | -0.03 | 0.06 | -0.09 | -0.13 |
| HR | 0.04 | 0.01 | -0.16 | -0.23 | -0.16 | 0.30 |
| ASDNN | -0.04 | -0.11 | -0.09 | -0.09 | -0.17 | -0.23 |
| SDANN | 0.10 | 0.05 | -0.24 | -0.14 | -0.19 | -0.38* |
| SDNN | 0.09 | 0.08 | -0.23 | -0.15 | -0.18 | -0.37* |
| RMSSD | -0.04 | -0.05 | -0.02 | -0.06 | -0.06 | -0.15 |

**Table S3.** Spearman correlation coefficients between ECG and CMR results for patients after early TOF repair; * = statistically significant results; QRSc - QRS duration corrected for heart rate; HR – heart rate; ASDNN - average of all 5-minute standard deviations of NN intervals; SDANN - standard deviation of the all five-minute averages; SDNN - standard deviation of all NN intervals ; RMSSD - square root of the mean squared differences of successive NN intervals; LVEF – left ventricular ejection fraction; RVEF – right ventricular ejection fraction; RVEDVI – right ventricular end-diastolic volume index; RVESVI - right ventricular end-systolic volume index; RVSVI - right ventricular stroke volume index; PRF – pulmonary regurgitation fraction.

| | LVEF | RVEF | RVEDVI | RVESVI | RVSVI | PRF |
|---|---|---|---|---|---|---|
| PQ | -0.10 | -0.30 | 0.09 | 0.15 | -0.17 | -0.29 |
| QRS | -0.03 | -0.61* | 0.56* | 0.54* | 0.33 | -0.11 |
| QRSc | -0.03 | -0.61* | 0.56* | 0.54* | 0.33 | -0.11 |
| QTc | -0.25 | -0.28 | -0.16 | -0.10 | -0.37 | -0.30 |
| HR | 0.03 | 0.14 | 0.02 | -0.01 | 0.20 | 0.52 |
| ASDNN | -0.05 | -0.33 | 0.11 | 0.21 | -0.02 | -0.25 |
| SDANN | -0.34 | -0.38 | 0.14 | 0.28 | -0.05 | -0.52* |
| SDNN | -0.40 | -0.24 | 0.30 | 0.47 | 0.07 | -0.43 |
| RMSSD | -0.04 | -0.41 | 0.14 | 0.24 | 0.05 | -0.06 |

**Table S4.** Spearman correlation coefficients between ECG and CMR results for patients after late TOF repair; * = statistically significant results; QRSc - QRS duration corrected for heart rate; HR – heart rate; ASDNN - average of all 5-minute standard deviations of NN intervals; SDANN - standard deviation of the all five-minute averages; SDNN - standard deviation of all NN intervals ; RMSSD - square root of the mean squared differences of successive NN intervals; LVEF – left ventricular ejection fraction; RVEF – right ventricular ejection fraction; RVEDVI – right ventricular end-diastolic volume index;

RVESVI - right ventricular end-systolic volume index; RVSVI - right ventricular stroke volume index; PRF – pulmonary regurgitation fraction.

| | **LVEF** | **RVEF** | **RVEDVI** | **RVESVI** | **RVSVI** | **PRF** |
|---|---|---|---|---|---|---|
| PQ | -0.25 | -0.29 | 0.10 | 0.14 | -0.05 | -0.18 |
| QRS | -0.14 | -0.11 | -0.04 | 0.03 | -0.07 | -0.25 |
| QRSc | -0.14 | -0.11 | -0.04 | 0.03 | -0.07 | -0.25 |
| QTc | -0.03 | 0.12 | <0.01 | 0.09 | 0.03 | <0.01 |
| HR | 0.02 | -0.09 | -0.22 | -0.26 | -0.30 | 0.20 |
| ASDNN | -0.11 | -0.11 | -0.13 | -0.14 | -0.19 | -0.27 |
| SDANN | 0.19 | 0.16 | -0.40 | -0.32 | -0.26 | -0.33 |
| SDNN | 0.18 | 0.21 | -0.40 | -0.34 | -0.24 | -0.35 |
| RMSSD | -0.05 | 0.02 | -0.07 | -0.18 | -0.09 | -0.27 |
